# Range expansion of the Bluetongue vector, *Culicoides imicola*, in continental France likely due to rare wind-transport events

**DOI:** 10.1038/srep27247

**Published:** 2016-06-06

**Authors:** Stéphanie Jacquet, Karine Huber, Nonito Pagès, Sandra Talavera, Laura E. Burgin, Simon Carpenter, Christopher Sanders, Ahmadou H. Dicko, Mouloud Djerbal, Maria Goffredo, Youssef Lhor, Javier Lucientes, Miguel A. Miranda-Chueca, Isabel Pereira Da Fonseca, David W. Ramilo, Marie-Laure Setier-Rio, Jérémy Bouyer, Christine Chevillon, Thomas Balenghien, Hélène Guis, Claire Garros

**Affiliations:** 1 Cirad, UMR15 CMAEE, 34398; INRA, UMR1309 CMAEE, 34398 Montpellier, France; 2 CNRS, Université de Montpellier, UMR 5290 Maladies Infectieuses & Vecteurs-Ecologie, Génétique, Ecologie, Contrôle (MIVEGEC), Montpellier, France; 3 IRD, UR 224 MIVEGEC, BP 64501, Agropolis, 34 394 Montpellier cedex 5, France; 4 INRA, UMR1309 CMAEE,34398; Cirad, UMR15 CMAEE, 34398 Montpellier, France; 5 Cirad, UMR15 CMAEE, 97170 Petit-Bourg, France; INRA, UMR1309 CMAEE 34398 Montpellier, France; 6 Centre de Recerca en Sanitat Animal (CReSA), UAB-IRTA, Campus de la Universitat Autònoma de Barcelona, Bellaterra (Cerdanyola del Vallès), Spain; 7 Met Office, Exeter, UK; 8 Vector-borne Viral Diseases Programme, The Pirbright Institute, Pirbright, UK; 9 West African Science Service on Climate Change and Adapted Land Use, Climate Change Economics Research Program, Cheikh Anta Diop University, Sénégal; 10 Institut National de la Médecine Vétérinaire (IMV), Laboratoire vétérinaire régional, Tizi Ouzou, Algeria; 11 Istituto Zooprofilattico Sperimentale dell’Abruzzo e del Molise ‘G. Caporale’, 64100 Teramo, Italy; 12 Office National de Sécurité Sanitaire des produits Alimentaires (ONSSA), Rabat, Morocco; 13 Faculdad de Veterinaria, University of Zaragoza (UZ), Zaragoza, Spain; 14 Laboratory of Zoology, University of Balearics (UIB), Palma de Mallorca, Spain; 15 CIISA, Faculdade de Medecina Veterinaria, Universidade de Lisboa (FMV-ULisboa), Lisboa, Portugal; 16 Entente interdépartementale pour la démoustication-Méditerranée (EID-Méd), Montpellier, France; 17 Institut Sénégalais de Recherches Agricoles (ISRA), Laboratoire National de l’Elevage et de Recherches Vétérinaires, Dakar, Sénégal

## Abstract

The role of the northward expansion of *Culicoides imicola* Kieffer in recent and unprecedented outbreaks of *Culicoides*-borne arboviruses in southern Europe has been a significant point of contention. We combined entomological surveys, movement simulations of air-borne particles, and population genetics to reconstruct the chain of events that led to a newly colonized French area nestled at the northern foot of the Pyrenees. Simulating the movement of air-borne particles evidenced frequent wind-transport events allowing, within at most 36 hours, the immigration of midges from north-eastern Spain and Balearic Islands, and, as rare events, their immigration from Corsica. Completing the puzzle, population genetic analyses discriminated Corsica as the origin of the new population and identified two successive colonization events within west-Mediterranean basin. Our findings are of considerable importance when trying to understand the invasion of new territories by expanding species.

Rapid shifts in the geographic distribution of arthropod species, including incursion into new regions, can have major ecological and economic impacts^1^. Among vectors of human or livestock-associated arboviruses, the most high profile recent examples of this phenomenon have been among the Aedenine mosquitoes, particularly *Stegomyia albopicta* (* = Aedes albopictus*) (Skuse), *Hulecoeteomyia japonica* ( = *Ae. japonicus*) (Theobald) and *Hu. koreica* ( = *Ae. koreicus*) (Edwards)^2^,^3^,^4^. Several lifecycle characteristics of these species facilitate their long-distance dispersal, most importantly their ability to diapause at egg stages that allows survival of periods of desiccation, and thus the exploitation of ephemeral water sources. This ability has allowed long-distance migration via global trade of plants and used tires^5^,^6^. Following migration, the establishment of new populations depends on the suitability of climatic and environmental conditions at the place of arrival. Rapid expansions in distribution associated with global trade have not generally been reported for the genus *Culicoides* Latreille (Diptera: Ceratopogonidae)^7^,^8^, although this has been hypothesized for *C. jamaicensis* Edwards^9^ and *C. belkini* (Wirth and Arnaud)^10^.

At a local scale, the distribution of major vector species of arboviruses can change according to environmental parameters and in turn influence disease distribution. An example is the primary Australian vector of bluetongue virus (BTV) *C. brevitarsis* Kieffer. The southern limit of distribution of this species on the central coastal region of New South Wales, Australia varies significantly with climatic variables and this in turn determines the limit of BTV and Akabane virus (AKAV) distribution each year^11^,^12^. Within Europe, it has been hypothesized that changes in the northern limits of *C. imicola* Kieffer in the Mediterranean basin have occurred and coincided with an unprecedented expansion of BTV in this region^13^,^14^,^15^. This hypothesis is challenged by recent genetic analyses that supported a long-time presence of *C. imicola* in the Mediterranean basin^16^,^17^. *Culicoides imicola* is the primary afrotropical vector species of BTV and African horse sickness virus (see review in^18^,^19^).

A key challenge for assessing the recent invasion hypothesis was that systematic data regarding the distribution of *C. imicola* prior to BTV incursions were rarely available. Entomological evidences of *C. imicola* presence in southern Europe (i.e. Balearic Islands, Italy, France, and continental Greece) dated from less than fifteen years^13^,^20^,^21^,^22^,^23^. No prior extensive surveys are available to determine if these territories were *C. imicola*-free before the 2000’s records. Since then, established populations of the species seem to have expanded their range by colonizing new habitats at the northern limit of the distribution range. Indeed, additional entomological surveys recorded the presence of the species in Catalonia, Spain^24^ and in Var department, France^13^. The observed low abundance of captured insects^25^ and physiological status^13^ suggest a recent northward expansion of these populations at the northern edge distribution of *C. imicola*.

In the Iberian Peninsula, the first recorded BTV outbreaks occurred in the 1960’s but confirmed presence of *C. imicola* populations was first reported in 1983 in Spain^26^ and soon afterwards in Portugal^27^. The latitude 40°N (i.e., that of Madrid) was then described as the northernmost limit of *C. imicola* with high abundances and continuous distribution characterizing the south-west quarter of the Iberian Peninsula^25^,^28^,^29^. *Culicoides imicola* was observed in the Balearic Islands in 2001–2002^20^. In 2002, the first detection of *C. imicola* in a coastal site of Catalonia (~41–42°N) marked a new incursion step toward the northern expansion of the species distribution^24^. The authors hypothesized that this establishment in Catalonia resulted from a windborne dispersal event from the Balearic Islands where *C. imicola* was found at high abundance^24^.

*Culicoides imicola* was recorded in Corsica in 2000^21^ and in the south-east of continental France (Var department) in 2003^13^. The establishment of *C. imicola* in the Var department was subsequently confirmed through extensive trapping surveys. There, the local expansion of the species distribution was estimated as 14.5 km/year and thought to be restricted by physical barriers and the limitation of both suitable larval habitats and suitable hosts for blood-feeding^13^.

The recent colonization and the establishment of populations of *C. imicola* in neighbouring countries have led to the question of whether incursions of this species will occur into mainland France^30^,^31^. Indeed, a recent ecoclimatic niche model predicted that additional habitats will become suitable for *C. imicola* colonization in Western Europe under climate change scenarios and predicted northward range expansion along the Spanish and French border^32^. As part of a risk assessment of this scenario the potential expansion of *C. imicola* from Catalonia to the south of France (Pyrénées-Orientales department) was therefore investigated from 2002 onwards^13^. Three individuals where captured in the Pyrénées-Orientales department in 2008, supporting the presence of *C. imicola* in the region^13^. This paper reports a new wave of range expansion and the establishment of *C. imicola* in the French mainland. We used a unique combination of population genetics and meteorological modelling of long-distance dispersal to trace the origin of these populations in relation to neighboring areas.

## Results

### Entomological surveys

**Figure 1:**
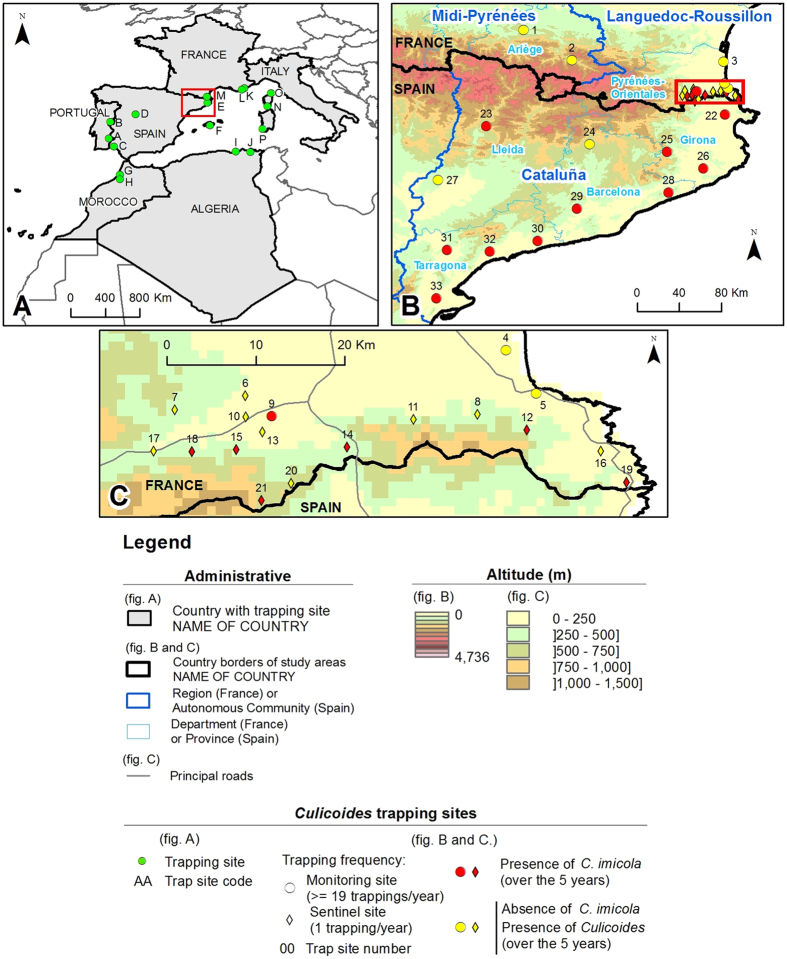
Sampling sites for population genetic analyses (**A**) and entomological surveys (**B, C**). Code sites are detailed in Table 1 and Supplementary Table 1. Maps were generated using ArcGIS software v10.2.2 (ESRI, Redlands, CA).

**Figure 2:**
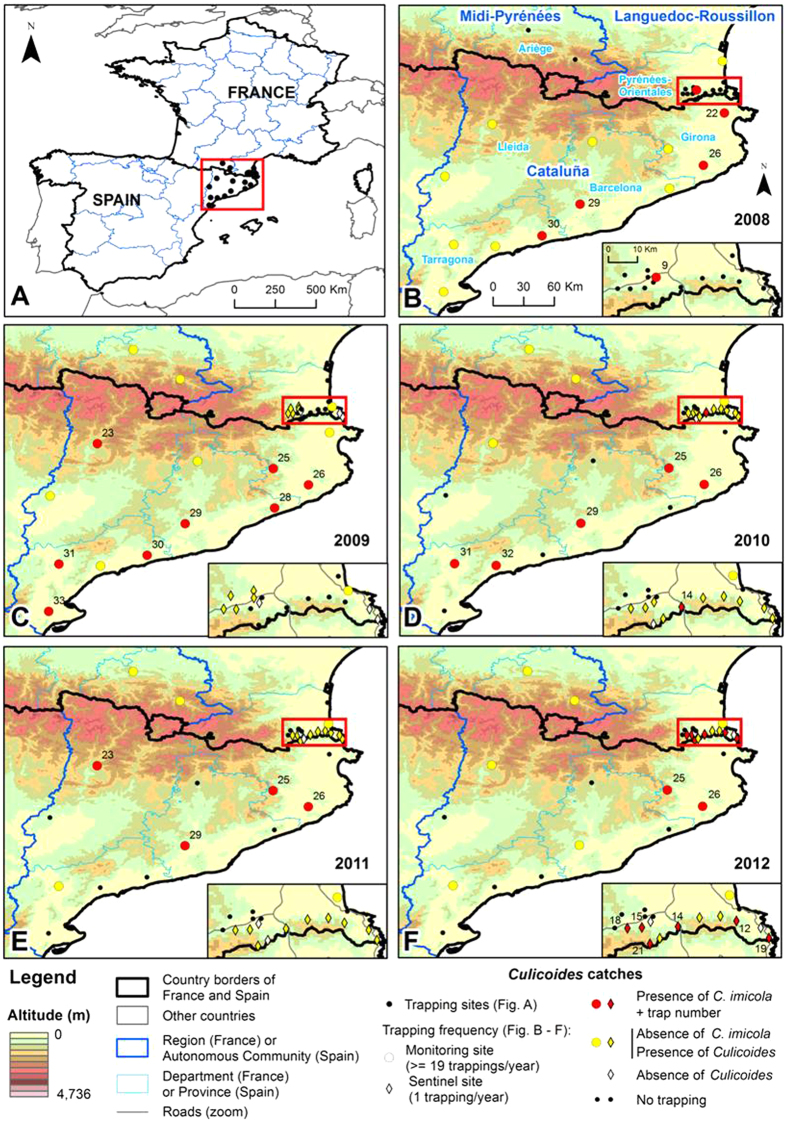
Presence/absence map of *C. imicola* in Pyrénées-Orientales and Catalonia from 2008 to 2012. Code sites are detailed in Supplementary Table 1. Maps were generated using ArcGIS software v10.2.2 (ESRI, Redlands, CA).

Within the entire study area, 2,375 nights of trapping were conducted from 2008 to 2012 at 15 sentinel sites along the French-Spanish border (with traps surveyed on a yearly basis) and at 18 monitoring sites in France and Spain (with traps surveyed on monthly or weekly bases) (Supplementary Table 1, Fig. 1). In Spain, *C. imicola* was collected at 10 of the 12 monitoring sites including three sites (Caldes, Piera and Susqueda) that had positive collections for four consecutive years (Supplementary Table 1, Fig. 2). In France, *C*. *imicola* was observed once at a monitoring site (St-Jean-Pla-de-Corts) during the five years of survey. *Culicoides imicola* was trapped in 6 out of the 15 sentinel sites with the highest records observed in 2012 (11 individuals/night). Maximum catches of *C. imicola* were relatively low at sentinel sites (<11 females/night) and monitoring sites (<24 females/night), except at two Spanish monitoring sites (Caldes and Susqueda) where more than 250 individuals were regularly collected (i.e., 250 individuals/night from 2009 to 2011), indicating established *C. imicola* populations (Fig. 2).

### Within population genetic diversity

We genotyped a total of 483 *C. imicola* adults sampled from 16 sites at nine microsatellite loci (Table 1, Fig. 1). In addition, a total of 1,107 base pairs of mitochondrial genes COI (474 bp) and CytB (633 bp) were sequenced for a subset of 132 individuals randomly selected among the successful genotyped insects. The analysis of the concatenated mitochondrial data provided a total of 31 haplotypes, among which two (H2 and H7) were dominant and distributed across the populations. The level of genetic variability within populations was comparable among sites (0.67 ± 0.12 ≤ H_d_ ≤ 0.95 ± 0.04) (Supplementary Table 2).

### Population genetic structure

Pairwise allelic tests based on 9 microsatellite loci failed to detect linkage disequilibrium among loci within sample-sites. All populations were in Hardy-Weinberg equilibrium with *F*_IS_ estimate ranging from −0.038 to 0.140 (Supplementary Table 3). Three models were used to test for recent genetic bottlenecks based on allele frequency data. While tests based on the IAM mutation model suggested potential signatures of past genetic bottlenecks in samples collected in Algeria, Var, Corsica, Pyrénées-Orientales and Sardinia, those based on the most realistic TPM and SMM mutation models were only significant for Roquebrune-sur-Argens (Var department, France) under the TPM model (Supplementary Table 3).

**Figure 3:**
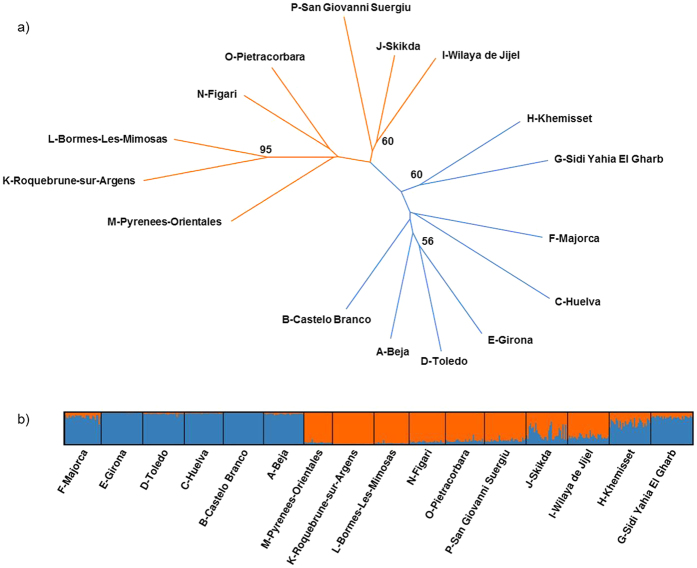
Microsatellite neighbor-joining tree and genetic clustering of *C. imicola* population samples. (**a**) The neighbor-joining tree is based on genetic distance of Cavalli-Sforza & Edwards (1967). Bootstrap values are calculated over 1,000 replicates (only values >60% are shown). (**b**) Each vertical line represents an individual, and each color represents a cluster. Individuals are grouped by sampling location: Algeria (Skikda, Wilaya de Jijel), Balearic Islands (Majorca), Continental France (Pyrénées-Orientales, Roquebrune-sur-Argens, Bormes-les-Mimosas), Continental Spain [Girona (Catalonia), Toledo, Huelva], Corsica (Figari, Pietracorbara), Morocco (Khemisset, Sidi Yahia El Gharb), Portugal (Beja, Castelo Branco), Sardinia (San Giovanni Suergiu).

Bayesian clustering analysis based on the microsatellite data identified two genetic groups, as ∆K was clearly maximum for K = 2 (∆K_max_ = 33), which corresponded to a “western cluster” including Morocco, Spain, Portugal and Majorca, and a “central cluster” consisting of Algeria, Corsica, Sardinia, Pyrénées-Orientales and Var departments (Fig. 3). This spatial genetic structure was consistent with that obtained with the microsatellite Neighbor-joining tree (Fig. 3). Interestingly, the Bayesian clustering analysis and microsatellite neighbor-joining tree suggested that Catalonian population (Girona) is genetically similar to all other continental Spanish populations. Likewise, midges from the Balearic Islands (Majorca) were most closely related to Moroccan and Continental Spanish populations.

**Figure 4:**
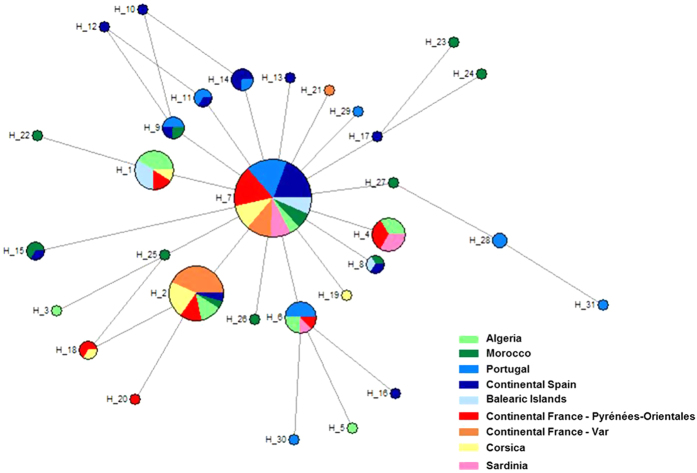
Median-joining haplotype network. The size of the circles is proportional to the number of individuals with that haplotype. The length of the branches separating haplotypes is proportional to the number of mutational steps between them.

**Figure 5:**
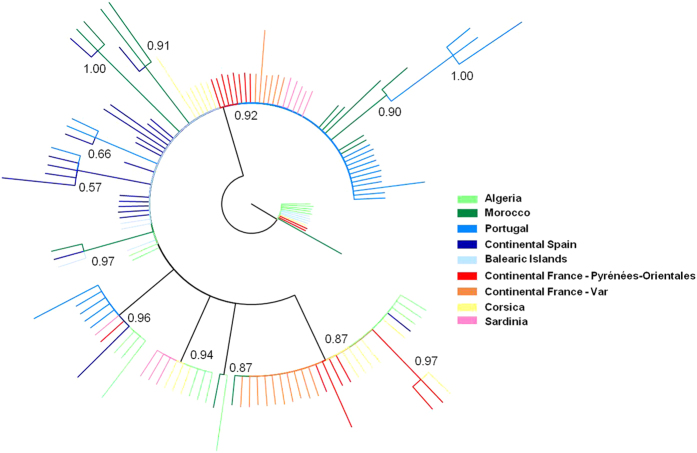
Mitochondrial Bayesian phylogenetic tree. Numbers represent the posterior probability and each color refers to a geographical region.

The spatial pattern was further supported by the median-joining mitochondrial haplotype network, which displayed strong genetic relationships between Pyrénées-Orientales, Sardinian, Algerian and French populations (Var department and Corsica) while the Spanish populations were genetically closer to those in Portugal and Morocco (Fig. 4). These genealogical relationships were also supported by the Bayesian phylogenetic tree (Fig. 5) and the mitochondrial pairwise F_ST_ values (Supplementary Table 4).

Considering the hierarchy of sampling, significant differentiation was detected between both genetic clusters (Fcluster-total = 0.016; P = 0.0001) but also within clusters (Fpopulations-clusters = 0.012; P = 0.0001).

Despite the geographical distances involved, pairwise F_ST_ estimates based on microsatellite data remained relatively low (F_ST_ ≤ 0.07; Table 2). The genetic differentiation tests were significant for several pairwise comparisons; and particularly when estimating among two populations that did not belong to the same genetic cluster inferred by STRUCTURE (Table 2).

### Genetic inference of colonization pathways

**Figure 6:**
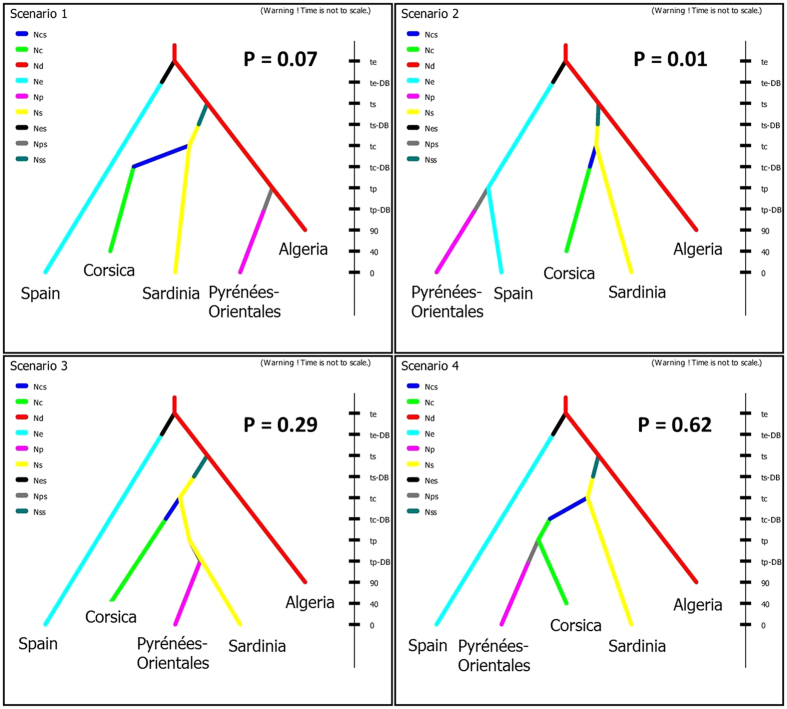
Graphical representation of the tested scenarios regarding colonization sources of *C. imicola* in the Pyrénées-Orientales. Microsatellite data were used and data were simulated using an approximate Bayesian computation (ABC) approach. The y-axis represents the time of events (not to scale), time 0 being the most recent sampling date. Nc, Ns, Nd, Ne and Np refer respectively to the effective population sizes, stable over the time, of the populations from Corsica, Sardinia, Algeria, Catalonia and Pyrénées-Orientales and Ncs, Nss, Nes and Nps refer to the effective number founder for Corsica, Sardinia, Catalonia and Pyrénées-Orientales populations. P refers to the probability obtained for each scenario. Details of all scenarios and parameters are shown in Supplementary Tables 5 and 6.

We tested the potential routes of colonization of *C. imicola* into Pyrénées-Orientales using ABC methods. Our results support the scenario involving Corsica as the source of Pyrénées-Orientales populations. More specifically, the most probable scenario entails a succession of three colonization events: the colonization of Sardinia by North African individuals, followed by the colonization of Corsica by Sardinian founders, and then colonization of Pyrénées-Orientales by Corsican emigrants (P = 0.62, 95% CI = [0.60–0.64]; Fig. 6, Supplementary Table 5). The type I and type II errors associated to this scenario were evaluated as 0.28 and 0.06, respectively (Supplementary Table 5). Model checking was carried out for the selected scenario. None of the summary statistics (used and unused for ABC inferences) displayed low probability (i.e. P < 0.05), indicating that the selected scenario fits well the observed data (Supplementary Table 6). This is also confirmed by a Principal Component analysis (PCA): PCA points simulated from the posterior predictive distribution grouped together closely and centered on the target point corresponding to the real dataset (Supplementary Fig. 1).

### Long-distance dispersal model outputs

**Figure 7:**
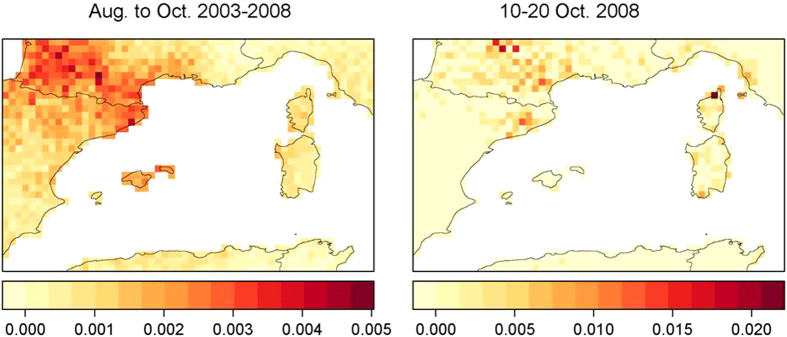
Source of winds potentially transporting *C. imicola* to the trap location in Pyrénées-Orientales. To generate this map, the NAME dispersion model was run in backwards mode for 36H each day from 1 August to 31 October for the period 2003 to 2008, using 30,000 particles (left panel). We also present the results of the simulations for the period 10–20 Oct. 2008 only (right panel). The probability of pixels as source points for Pyrénées-Orientales was calculated as the total number of particles received in each pixel from the individual daily simulations divided by the total particles received by all the grid cells not located over the sea for each time period. Maps were generated using R software v3.2.2.

The areas of the study region most likely to have been source regions of windborne *C. imicola* were assessed using the NAME model. The resulting air frequency map shows that air arriving at the entry point (Saint-Jean-Pla-de-Corts, site 9 in Fig. 1) during the full studied time period (1st of August to 31st of October 2003 to 2008) frequently came from north-eastern Spain and Balearic Islands (Fig. 7, left panel). At some periods however, rare wind-borne transport events made northern Corsica (Fig. 7, right panel) the most likely source for *C. imicola*. Air only occasionally arrived at the trap site from Corsica, other parts of southern France, parts of Italy or the northern coast of Africa within the 36 hour time limit.

The individual trajectory maps described a similar pattern. Full 36-hour back-trajectories for all particles together are presented for each day during the full observation period in supplementary file video clip 1.

## Discussion

This study reports a second incursion of *C. imicola* in continental France beyond the apparent northern edge of the species distribution. By using a combination of standard population genetics and approximate Bayesian computation methods, we were able to determine that this newly discovered population was not closely related to the nearby (~80 km south) populations settled in Catalonia. Instead, the newly settled *C. imicola* population was shown by both nuclear and mitochondrial genetic loci to be closely related to far more distant populations (360 to 1,000 km east or south-east) in the Var department, Corsica, Sardinia and Algeria. Corsica was further supported as the most likely source of introduction by the ABC analyses, suggesting that establishment of *C. imicola* in Pyrénées-Orientales could have occurred through long-distance dispersal from abundant populations in the island (>500 km from the mainland sampling site). However, other potential population sources such as smaller populations in the Var department or yet undiscovered populations (despite entomological surveillance in this area) between these on the southern coast of France cannot be totally discounted.

Research on the dispersal activity of *Culicoides* is divided into two main areas of focus. Long-distance semi-passive flights on prevailing winds over water bodies have been investigated as a means of both predicting and retrospectively identifying sources of incursions (see ref. 33 for a review). In the current study, we used NAME to simulate the potential for *Culicoides* dispersal to Pyrénées-Orientales and found that trajectories centered primarily on directly surrounding areas, including north-eastern Spain and Balearic Islands. These trajectories also sometimes comprised simulated particles originating from distant areas including northern Corsica and Sardinia, suggesting that midges’ dispersal from these sources were possible, but related to rare wind-transport events during the period of abundance of this species.

Although the Pyrénées is a limited elevated mountainous chain, it appears to shape the *C. imicola* population genetic structure more than expected. NAME has been most successfully applied to trajectory simulations over water bodies and would require adaptations to be applicable for local-scale movements over land due to the influence of topographical complexity. Abundance of population sources is also a key factor to take into account. The probability to reach a point by long-distance dispersal depends on the number of active midges that will spread and then survive during transportation. The low abundances observed in Catalonia (maximum catch ~12,000 individuals per night), Balearic Islands (mean number 5–26 individuals per night per trap)^34^ and the Var department (>100 individuals per night and maximum catch >4,001 individuals per year)^13^ compared to Sardinia and Corsica (30,000–100,000 individuals per night)^13^,^35^, suggest that these populations unlikely to act as a seed source. A combination of high abundance and favorable winds may support the dispersion of midges from Corsica reaching Pyrénées-Orientales.

Combining the results provided by the NAME model and genetics approach suggests that long-distance dispersal events contribute to *C. imicola* introduction and colonization of new areas. Our genetic analyses also allowed the assessment of the origin of the Catalonian populations. We discounted the previous hypothesis of the Catalonian population being sourced from the Balearic Islands via windborne dispersal^24^. The microsatellite neighbor-joining tree as well as the Bayesian clustering analysis indicates instead that the Catalonian population is genetically closer to any other continental Spanish populations than to the insular Balearic population. Moreover, North-Africa appears as a much more likely source of the Balearic populations than Sardinia, which hosts *C. imicola* populations closely related to the French ones.

A second major area of current research in *Culicoides* flight is active dispersal in random directions that can reach 2.21 km daily. This has been investigated recently in northern Europe using capture, mark recapture (CMR) techniques based on fluorescent dusts^36^,^37^ or immunomarking^38^. Historically, the maximum distance that a recapture has made in this type of study is at 6 km in the peculiar case of *Culicoides mohave* Wirth in the USA^39^, a species which breeds in desert areas. Interestingly, the speed of colonization recorded for *C. imicola* populations over land in the Var region appears to be limited^13^. This may be a consequence of low population density in the Var region^13^ and landscape barriers to population spread. The inland limit of *C. imicola* in the Var region in France appears to be restricted by the South Alps. This is consistent with intensive surveys at several sites along the French Mediterranean coast that failed to detect *C. imicola* outside this region between 2002 and 2010^13^. Nonetheless, more targeted surveys of the southern coast of France for further *C. imicola* populations would be useful in ensuring that the range of this species has not been overlooked in these areas. The investigation of landscape barriers to dispersal of *Culicoides* remains a relatively poorly investigated area. Studies of local-scale landscape ecology could fall below the resolution of genetic techniques, such as microsatellite analysis. In this regard, the use of genome-wide single nucleotide polymorphisms (SNPs), accessed via next-generation sequencing methods, may provide greater resolution at a local scale and advance our understanding of population processes^40^. This may in turn enable improvements in the accuracy of predictive models for *Culicoides* dispersal over land through integration of meteorological, landscape and activity-based parameters^33^.

The influence of globalized transport on *Culicoides* dispersal and colonization of new areas remains poorly understood. The introduction of infected *Culicoides* into Europe via trade routes has been cited as one of many potential points of entry of arboviruses, but direct data remains extremely limited^41^. *Culicoides* have been recorded as being present at low number on aircraft (number unknown)^8^ or ships (~1 adult/ship)^7^, and such estimates are probably conservative due to the logistical challenges of sampling. Recent modeling analyses showed that the risk of introduction of infected *Culicoides* via transport and trade networks to Spain from other European countries is low^42^,^43^ although these studies are largely based upon very poorly defined parameters. In the current study, Corsica, the Var department, Algeria and Sardinia share no major ruminant or equine trade links with Pyrénées-Orientales, suggesting that windborne dispersal remains the most likely migration means among these localities.

Except in two sites in Spain, the observed *C. imicola* abundance remains very low in the French and Spanish study sites, and no massive expansion was observed, as was observed in the Var department^13^. The role of adverse meteorological conditions (wind, rain) on *Culicoides* population dynamics has been described and may have influenced our results on species abundance. This probably explains the overall low number of *Culicoides* collected in 2009 in France (the week of prospection was particularly rainy and windy). The relatively limited abundance in monitoring sites compared to other parts of the *C. imicola* distribution area e.g.^44^ could be explained by climatic conditions that might be less suitable in this region and/or by the fact that this region is presumably the northern edge of *C. imicola* distribution.

Our work highlights that observation bias related to entomological surveys could lead to misinterpretation of routes and population sources of colonization, especially when the targeted species is a small size and highly passive dispersive species. Our results are consistent with the hypothesis of an introduction by winds, into Pyrenées-Orientales from Corsica. The combination of independent approaches using population genetic analysis and modeling of long-distance dispersal of *Culicoides* confirm the importance of windborne transport for the spread of exotic species and infected females. Facing numerous signals of long dispersal of *Culicoides* populations, one should now estimate the frequency of these events, especially when outbreaks are declared in Northern Africa while free statuses are maintained in continental areas.

## Methods

### Entomological surveys and species identification

Thirty-three sites in France and Spain were sampled for *Culicoides* from 2008 to 2012 (Fig. 1). Two levels of sampling effort can be distinguished (Supplementary Table 1, Figs 1 and 2): monitoring sites were used in the national surveillance network for *Culicoides* populations in the two countries and operated throughout the year on a weekly or monthly basis; sentinel sites in the Pyrénées-Orientales department (France) were visited once a year to survey *C. imicola* expansion from the 2008 detection point (Supplementary Table 1, Fig. 2). Surveys of sentinel sites were carried out during early autumn (September/October) to match the abundance peak of *C. imicola*^13^. Sampling was carried out using ultra-violet light-suction traps (Onderstepoort design) in France and miniature CDC black light traps in Spain, in close proximity to animal shelters containing sheep, cattle or horses and operated from dusk to dawn. Collections were stored in 90% ethanol prior to species identification. Morphological identification of *C. imicola* within samples was carried out to species level using wing pattern^21^,^45^.

### Population genetics

#### DNA extraction and amplification

A total of 483 *C. imicola* individuals from 16 localities in North Africa and south-western Europe were used for microsatellite analyses, and a portion of the mitochondrial genes Cytochrome oxydase subunit I (COI) and Cytochrome b (CytB) were sequenced for 132 successful genotyped individuals (Table 1, Fig. 1). Microsatellite data as well as COI and CytB sequences from eight of the localities were previously published in^16^ (see details in Table 1). Genomic DNA was extracted from single adult *C. imicola* using a NucleoSpin96 Tissue Kit (Macherey-Nagel, Duren, Germany) according to the manufacturer’s instructions. Nuclear genotyping was conducted at 9 microsatellite markers previously developed for *C. imicola* by Mardulyn *et al.*^17^ (Supplementary Table 7) and following the protocol described in^16^. Insects were sequenced for the mitochondrial genes COI and CytB using the primers C1J1718/C1N2191 and CytB_12329F/CytB_13038R, respectively, as described in^16^.

#### Sequence analyses

All the sequences were edited and aligned with ClustalW algorithm implemented in the software GENEIOUS v.6.0.5 (Biomatters, www.geneious.com). COI and CytB data sets were analysed separately and showed the same pattern but with a lower resolution. We thus combined COI and CytB data for all analyses.The genetic diversity was estimated by computing the number of haplotypes (H), haplotype diversity (H_d_) and nucleotide diversity (π) using DNASP v.5^46^. The relationships and the geographical distribution of genetic variation among sites were explored with a median-joining network^47^ conducted in Network v.4.6.1.2 (www.fluxus-engineering.com) on the concatenated COI and CytB dataset. Genealogical relationships were further investigated by a Bayesian phylogenetic inference as implemented in MRBAYES v.3.2.2^48^. The software JMODELTEST v.2.1.3^49^ was used to assess the best-fit substitution model based on the Akaike Information Criterion (AIC). The phylogenetic tree was estimated after 1 million generations of four Markov chains ran twice and sampled every 100 generations. Chain convergence was checked with Tracer v.1.6 software^50^ and the first 2,500 generations were discarded as burn-in phase. Finally, population structure was assessed by computing pairwise F_ST_ values between populations.

#### Microsatellite analyses

The genotype of each individual was characterized with the software GeneMapper^®^ 4.0 (AppliedBiosystems). Linkage disequilibrium between all pairs of loci was tested using FSTAT v2.9.3.2^51^. Within-population departure from Hardy-Weinberg proportions was investigated by estimating the inbreeding coefficient (F_IS_). The significance of this estimator was assessed by randomizing alleles among individuals within samples (10,000 permutations). To visualize the genetic relationships between the sampled sites, we constructed a neighbor-joining (NJ) tree^52^ based on the pairwise genetic distances of Cavalli-Sforza and Edwards using the software POPULATIONS v.1.2.30 (http://bioinformatics.org/~tryphon/populations/). The robustness of nodes was evaluated by carrying out 1,000 bootstrap replicates.

The Bayesian approach implemented in STRUCTURE v.2.3.3^53^ was used to infer spatial genetic structure. We assumed an admixture model with correlated allele frequencies^54^ and used the sampling locations (Locprior model) as priors’ information^55^. For each value of the number (K) of clusters set between 1 and 14 (number of sampled sites), we performed 10 independent runs of 10^6^ Markov chain Monte Carlo (MCMC) iterations with a burn-in of 10^5^. The most probable number of clusters was inferred using ∆K method^56^.

The relative importance of the genetic clusters previously inferred by STRUCTURE and the populations in differentiation was assessed with the multilocus hierarchical F-statistics Fpopulations-clusters and Fclusters-total, respectively. This analysis was performed with Hierfstat package^57^. These tests were based on 10,000 permutations of either *Culicoides* genotypes among populations and within clusters (H0: ‘Fpopulations-cluster = 0’), or populations among clusters (H0: ‘Fclusters-total = 0′). Genetic differentiation among samples was further assessed through the Weir and Cockerham^58^’s unbiased estimates F_ST_ and the significance was tested using the exact G test over 10,000 permutations of genotypes among samples as implemented in FSTAT v2.9.3.2^51^.

In populations that have undergone a sharp decrease in effective population size, the loss of alleles is faster than the decline of genetic diversity (H_S_). This results in an increase of heterozygosity across loci. The program BOTTLENECK allows testing of this event in a representative sample of individuals^59^. It has been shown that past bottleneck events will be detected with a high degree of sensitivity using the Infinite Allele Mutation (IAM) model, moderately with the two-phase model (TPM) and dimly with the Stepwise Mutation Model (SMM)^60^. We therefore performed the unilateral Wilcoxon test under the three proposed mutation models^60^. For the TPM model the proportion of SMM was set to 70% and the variance to 30 (default values). The significance was assessed by performing 10,000 replicates.

#### Inference of colonization pathways

Microsatellite data were used to investigate the source of *C. imicola* individuals in Pyrénées-Orientales (Continental France) and test hypotheses regarding the observed genetic clusters using approximate Bayesian computation (ABC). Our hypotheses addressed four potential sources of *C. imicola*: Catalonia, Corsica, Sardinia or Algeria. We tested four demographic scenarios presented in Supplementary Table 4 and Fig. 6 with DIYABC software v.2.0.4^61^,^62^. Data were simulated under demographic, historical and mutational parameter values used as priors’ information given in Supplementary Table 8. We assumed 10 generations per year^63^, a divergence time starting 40 generations ago with 10,000 generations of uncertainty, and a mutation rate ranging from 10^−6^ to 10^−4^. Genetic variation within and between populations was summarized using a set of statistics implemented in DIYABC including the mean number of alleles, the mean expected heterozygosity^64^, the mean allelic size variance, the Garza-Williamson’s M (mean ratio of the number of alleles over the range of allele sizes)^65^, pairwise F_ST_ values^66^ and the classification index (mean individual assignment likelihood)^67^. The posterior probabilities for each of the competing scenarios were calculated by a polychotomous logistic regression^61^,^62^ on 1% of the simulated data sets similar to the observed data set. Confidence in the selected scenario was evaluated by analyzing 100 simulated pseudo-observed data sets (pods) with the same number of loci and individuals as our data set. The parameter values drawn from prior distribution (Supplementary Table 8) and LDA-transformed summary statistics were used to calculate type I and II errors. These latters refer to the probability of excluding the selected scenario when it is true and the probability of selecting the scenario when it is false, respectively. Mean type II error was calculated over the competing scenarios. Finally, we assessed the goodness of fit of the selected scenario by using the model checking option of DIYABC software^61^, which allows evaluating whether the selected scenario and associated posteriors distributions match well with the observed genetic data of *C. imicola*. As recommended by Cornuet *et al.*^61^, we used as test statistics the DIYABC summary statistics not used for model selection in previous ABC treatments. Because this analysis may suffer from non-independence between the summary statistics, we also performed a principal component analysis (PCA) in the space of the summary statistics.

### Model of long-distance biting midge dispersal

Possible windborne incursion of *C. imicola* into the study region were assessed using the Numerical Atmospheric-dispersion Modelling Environment (NAME) Lagrangian model, designed to simulate the release, transport, mixing and transformation of airborne gases or particulates and their subsequent depletion or removal from the atmosphere^68^. The release and dispersion of hundreds of thousands of model particles allows for representation of the stochastic nature of the atmosphere. The motions of the particles are determined by the ambient three-dimensional wind flow with a random component superimposed to simulate turbulence. The underlying meteorological data necessary to drive the dispersion model was taken from the UK Met Office’s Unified Model^69^. For Aug to Oct 2003 to 2008, the horizontal resolution of the Unified Model over Europe was 12 km with a temporal resolution of 1 hour.

NAME was chosen over other dispersion models as it has been previously used to describe wind-borne incursion events that correlate with the timing and location of outbreaks of BTV in Europe^33^,^70^ and compared favorable against another complex dispersion model, MATCH, for outbreaks in Sweden^71^. Simpler wind trajectory models have also been used to assess transport of *Culicoides* in the atmosphere^72^,^73^,^74^. These studies only follow the path taken by one trajectory at very low temporal and spatial resolution (typically 6 hourly at a horizontal resolution of 0.25° × 0.25°) and therefore cannot account for the stochastic nature of the atmosphere. Other Lagrangian particle-dispersion models are also available, such as the HYSPLIT model used by^75^ to assess incursions of *Culicoides* into Australia. However the underlying meteorological data that is freely available to use with this model for our study period and region is only available at 3-hourly intervals with a horizontal resolution of 1°. These scales would not be adequate for modelling the transport of *Culicoides* within the Mediterranean basin.

In this study, the model was run in backwards mode to simulate the source of winds potentially transporting *C. imicola* to the trap location in Pyrénées-Orientales. In backwards mode the wind direction is reversed and the model steps backwards through time. Saint-Jean-Pla-de-Corts (Site 9, Fig. 1) was selected as the entry point in 2008 as this was the first location where *C. imicola* was recorded. The period from 1 August to 31 October covering the peak of *C. imicola* abundance was assumed to be the period most likely for an introduction to the trap location and we thus modeled particles movement for this period from 2003 to 2008. A large number of model particles (30,000) were released in the model from the trap location for each day in the time window and tracked backwards for 36 hours (assumed to be the maximum flight time for *C. imicola*). At the end of each day’s simulation period the total number of particles present in each box of a 0.25° × 0.25° grid defined over the region were calculated. The greater the number of particles present in each grid box, the greater the proportion of air arriving at the trap site from that source. To assess where air most frequently arrived from during the likely introduction window, the relative probabilty of pixels as source points for Pyrénées-Orientales was mapped throughout the region (Fig. 7). It was calculated as the total number of particles received in each grid cell from the individual daily simulations divided by the total number of particles received by all the grid cells not located over the sea (which cannot be a source for culicoides populations) for a given period of time. In addition individual trajectories taken by 100 particles on each day in the time window were also calculated and examined to analyse the routes taken by individual air streams. Clustering of trajectories due to a dominant wind pattern can be identified, with some individual trajectories being taken in a very different direction due to turbulence or a separate synoptic system. In Fig. 7, we illustrate the fact that in some periods (e.g. 10–20 Oct. 2008), the pattern is very much different from the general pattern (mean values for 2003 to 2008). To illustrate the modeling process, supplementary file video clip 1 presents the 36-hour trajectories with a one hour time step for days 11/09/2008 and video clip 2 shows the full 36-hour back-trajectories for all particles together for each day during the full observation period (2003 to 2008).

## Additional Information

Accession codes: The COI and CytB sequences generated in this study were deposited in GenBank under accession numbers KX083462 - KX083520 and KX083403 - KX083461.

**How to cite this article**: Jacquet, S. *et al.* Range expansion of the Bluetongue vector, *Culicoides imicola*, in continental France likely due to rare wind-transport events. *Sci. Rep.* **6**, 27247; doi: 10.1038/srep27247 (2016).

## Supplementary Material

Supplementary Video

Supplementary Information

## Figures and Tables

**Table 1:** Geographical locations, sampling dates and number of *C. imicola* individuals typed for the population genetics analysis.

Country	Location	Code	Collection year	N_mic_	N_MtDNA_
Algeria	Skikda^16^	J	2003	32	9
	Wilaya de Jijel	I	2003	32	8
Morocco	Khemisset	H	2002	32	6
	Sidi Yahia El Gharb^16^	G	2004	32	8
Portugal	Castelo Branco^16^	B	2010	31	8
	Beja	A	2010	31	6
Balearic Islands, Spain	Majorca^16^	F	2012	28	8
Continental Spain	Girona, Catalonia	E	2012	32	6
	Toledo, Castilla-La-Mancha	D	2012	32	8
	Huelva, Andalusia^16^	C	2012	30	8
Continental France	Pyrénées-Orientales	M	2012	22	17
	Roquebrune-sur-Argens	K	2008	32	8
	Bormes-les-Mimosas^16^	L	2008	27	8
Corsica, France	Figari	N	2008	28	8
	Pietracorbara^16^	O	2008	30	8
Sardinia, Italy	San Giovanni Suergiu^16^	P	2012	32	8

N_mic_ and N_MtDNA_ refer, respectively, to the number of individuals typed for microsatellite analyses and the number mitochondrial sequences obtained. In Pyrénées-Orientales, due to the very small number of individuals collected in each trap, the sample consists of a mix of samples collected in 2012 in different locations (Reynes, Maureillas, Ceret ; see Supplementary Table 1).

**Table 2:** Pairwise F_ST_ values between *C. imicola* populations samples.

Western cluster								Central cluster							
Locations	E-Girona	D-Toledo	C-Huelva	B-Castelo Branco	A-Beja	H-Khemisset	G-Sidi Yahia El Gharb	M-Pyrénées-Orientales	K-Roquebrune-sur-Argens	L-Bormes-les-Mimosas	N-Figari	O-Pietracorbara	P-San Giovanni Suergiu	J-Skikda	I-Wilaya de Jijel
F-Majorca	0.0095	0.0113	0.0086	0.0057	−0.0015	0.0057	−0.0017	0.0242	0.0375	0.0320	0.0126	0.0054	0.0392	0.0118	0.0158
E-Girona		−0.0033	0.0177	−0.0002	−0.0002	0.0089	0.0093	0.0341	0.0620	0.0446	0.0326	0.0203	0.0352	0.0265	0.0308
D-Toledo			0.0233	−0.0040	−0.0038	0.0051	0.0089	0.0236	0.0570	0.0366	0.0276	0.0168	0.0336	0.0250	0.0340
C-Huelva				0.0066	0.0127	0.0243	0.0152	0.0295	0.0627	0.0643	0.0236	0.0228	0.0423	0.0244	0.0309
P-Castelo Branco					−0.0012	0.0033	0.0023	0.0185	0.0529	0.0430	0.0118	0.0104	0.0172	0.0102	0.0244
P-Beja						0.0079	0.0056	0.0164	0.0485	0.0342	0.0143	0.0082	0.0262	0.0155	0.0220
H-Khemisset							−0.0074	0.0142	0.0405	0.0322	0.0139	0.0145	0.0228	0.0082	0.0251
G-Sidi Yahia El Gharb								0.0144	0.0290	0.0250	0.0087	0.0068	0.0286	0.0021	0.0193
M-Pyrénées-Orientales									0.0231	0.0237	−0.0003	0.0110	0.0149	0.0170	0.0097
K-Roquebrune-sur-Argens										0.0100	0.0249	0.0171	0.0527	0.0342	0.0279
L-Bormes-les-Mimosas											0.0247	0.0210	0.0472	0.0305	0.0259
N-Figari												−0.0045	0.0099	0.0043	0.0090
O-Pietracorbara													0.0219	0.0056	0.0064
P-San Giovanni Suergiu														0.0044	0.0109
J-Skikda															0.0002

F_ST_ values are grouped according to the genetic clusters inferred by STRUCTURE v.2.3.3: western cluster (Spain, Portugal, Morocco) and central cluster (Algeria, Continental France, Corsica). The first letter in front of each location name refers to corresponding country: A–B, Portugal; C–E, Continental Spain; F, Balearic Islands; G–H, Morocco; I–J, Algeria; K–M, Continental France; N–O, Corsica; P, Sardinia. Significant values, at the adjusted nominal level (5%) for multiple comparison of 0.000476, are highlighted in bold.
