# Supplementary material for: Range expansion of the Bluetongue vector, Culicoides imicola, in continental France likely due to rare wind-transport events
Source: Sci Rep. 2016 Jun 6;6:27247. doi: 10.1038/srep27247 (PMC4893744; doi:10.1038/srep27247)
Supplement: Supplementary Information [file srep27247-s2.pdf]

Range expansion of the Bluetongue vector, *Culicoides imicola*, in continental France likely due to rare wind-transport events

Stéphanie Jacquet[1, 16, 17*], Karine Huber[2], Nonito Pagès[3, 4], Sandra Talavera[4], Laura E. Burgin[5], Simon Carpenter[6], Christopher Sanders[6], Ahmadou H. Dicko[7, §], Mouloud Djerbal [8, §], Maria Goffredo[9, §], Youssef Lhor[10, §], Javier Lucientes[11, §], Miguel A. Miranda-Chueca[12, §], Isabel Pereira Da Fonseca[13, §], David W. Ramilo[13, §], Marie-Laure Setier-Rio[14, §], Jérémy Bouyer[1, 15], Christine Chevillon[16, 17], Thomas Balenghien[1], Hélène Guis[1], Claire Garros[1*]

[1] Cirad, UMR15 CMAEE, 34398 Montpellier, France; INRA, UMR1309 CMAEE, 34398 Montpellier, France.[2] INRA, UMR1309 CMAEE, 34398 Montpellier, France; Cirad, UMR15 CMAEE, 34398 Montpellier, France. [3] Cirad, UMR15 CMAEE, 97170 Petit-Bourg, France; INRA, UMR1309 CMAEE 34398 Montpellier, France. [4] Centre de Recerca en Sanitat Animal (CReSA), UAB-IRTA, Campus de la Universitat Autònoma de Barcelona, Bellaterra (Cerdanyola del Vallès), Spain. [5] Met Office, Exeter, UK. [6] Vector-borne Viral Diseases Programme, The Pirbright Institute, Pirbright, UK. [7] West African Science Service on Climate Change and Adapted Land Use, Climate Change Economics Research Program, Cheikh Anta Diop University, Dakar-Fann, Sénégal. [8] Institut National de la Médecine Vétérinaire (IMV), Laboratoire vétérinaire régional, Tizi Ouzou, Algeria. [9] Istituto Zooprofilattico Sperimentale dell'Abruzzo e del Molise 'G. Caporale', 64100 Teramo, Italy. [10]Office National de Sécurité Sanitaire des produits Alimentaires (ONSSA), Rabat, Morocco. [11] Faculdad de Veterinaria, University of Zaragoza (UZ), Zaragoza, Spain.[12] Laboratory of Zoology, University of Balearics (UIB), Palma de Mallorca, Spain. [13] CIISA, Faculdade de Medecina Veterinaria, Universidade de Lisboa (FMV-ULisboa), Lisboa, Portugal. [14] Entente interdépartementale pour la démoustication-Méditerranée (EID-Méd), Montpellier, France. [15] Institut Sénégalais de Recherches Agricoles (ISRA), Laboratoire National de l'Elevage et de Recherches Vétérinaires, Dakar, Sénégal. [16] CNRS, Université de Montpellier, UMR 5290 Maladies Infectieuses & Vecteurs-Ecologie, Génétique, Ecologie, Contrôle (MIVEGEC). [17] IRD, UR 224 MIVEGEC, BP 64501, Agropolis, 34 394 Montpellier cedex 5, 25 IRD, (INTERTRYP), UMR177 IRD-CIRAD, TA A-17/G, Campus International de Baillarguet, Montpellier, France.

*Corresponding authors

*Cirad, UMR Contrôle des Maladies Animales Exotiques et Emergentes, Campus International de Baillarguet, TA-A15/G, 34398 Montpellier cedex 5, France, stephanie.jacquet@cirad.fr, claire.garros@cirad.fr

[$] by alphabetical order.

Supplementary Information

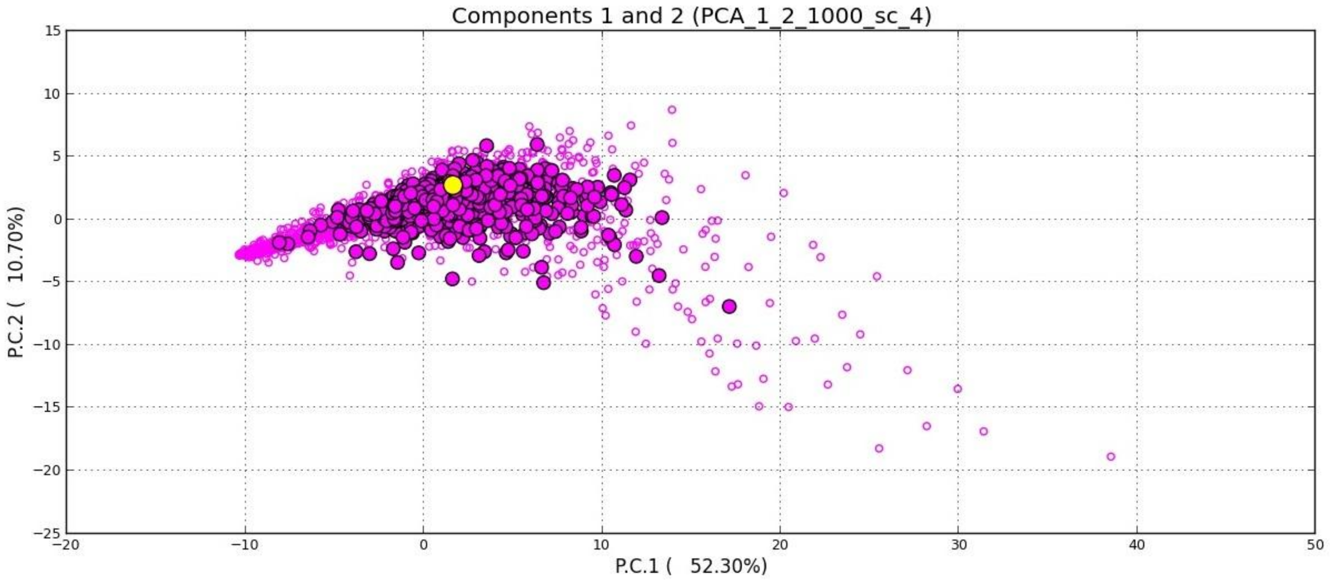

Supplementary Figure 1 | Principal component analysis (PCA) in the space of summary statistics performed under the most probable scenario (Scenario 4). The observations are the simulated datasets and the variables are the summary statistics. Small pink circles correspond to simulated statistics with parameters drawn from the prior distributions, the large pink dots correspond to the simulated datasets drawn from the posteriors distributions and the yellow dot represents the real *C. imicola* data set.

Supplementary Video clip 1 | NAME simulations of individual trajectories. Full 36-hour back-trajectories for all particles together are presented for each day during the full observation period. Maps were generated using ArcGIS software v10.2.2 (ESRI, Redlands, CA).

Supplementary Table 1 | Details of the entomological surveys realized in Spain and in France from 2008 and 2012.

		2008					2009					2010					2011					2012				
				Maximum *C. imicola* collection					Maximum *C. imicola* collection					Maximum *C. imicola* collection					Maximum *C. imicola* collection					Maximum *C. imicola* collection		
**code**	Location	Nb night collection during the year	Total nb of *Culicoides* collected	Collection date (DD /MM/YY)	Nb of *C. imicola*	Nb of other *Culicoides* collected	Nb night collection (DD /MM/YY)	Total nb of *Culicoides* collected	Collection date (DD /MM/YY)	Nb of *C. imicola*	Nb of other *Culicoides* collected	Nb night collection during the year	Total nb of *Culicoides* collected	Collection date (DD /MM/YY)	Nb of *C. imicola*	Nb of other *Culicoides* collected	Nb night collection during the year	Total nb of *Culicoides* collected	Collection date (DD /MM/YY)	Nb of *C. imicola*	Nb of other *Culicoides* collected	Nb night collection during the year	Total nb of *Culicoides* collected	Collection date (DD /MM/YY)	Nb of *C. imicola*	Nb of other *Culicoides* collected
**Monitoring sites**																										
**Spain**																										
**23**	Aramunt	52	590	-	0	-	52	375	02/07/2009	2	5	52	7,594	-	0	-	52	6,801	25/08/2011	1	38	52	3,188	-	0	-
**26**	Caldes	52	30,905	07/08/2008	129	699	52	9,801	10/09/2009	1,464	37	52	6,786	16/09/2010	11,627	358	52	22,817	01/09/2011	6,344	194	52	1,705	11/10/2012	739	0
**31**	Garcia	52	297	-	0	-	52	163	03/09/2009	1	0	52	184	20/05/2010	1	18	52	843	-	0	-	52	236	-	0	-
**29**	Piera	52	1,303	11/09/2008	1	28	52	2,001	08/10/2009	4	340	52	50,026	09/09/2010	7	5,207	52	8,551	03/11/2011	1	214	26	729	-	0	-
**25**	Susqueda	52	24,702	-	0	-	52	17,904	16/07/2009	296	4,654	52	40,123	01/07/2010	283	4,250	52	26,218	26/05/2011	279	4,103	26	3,604	24/05/2012	2	420
**24**	Avia	52	30	-	0	-	52	729	-	0	-															
**32**	Montbrio	52	10	-	0	-	52	64	-	0	-	19	3	29/04/2010	1	2										
**27**	Alguaire	52	29	-	0	-	52	110	-	0	-															
**33**	Amposta	52	76	-	0	-	52	667	31/12/2009	1	16															
**30**	Bonastre	52	44	21/08/2008	4	4	52	63	15/10/2009	24	12															
**28**	Sant Iscle	52	25,763	-	0	-	52	2,391	15/10/2009	3	54															

**22**	Vilanova Muga	52	6,486	27/03/2008	1	13	52	7,887	-	0	-															
**France**																										
**9**	**St Jean Pla de Corts**	20	144	**07/10/2008**	**3**	20																				
**2**	Vebre						25	8,972	-	0	-	26	23,788		0		25	2,305		0		27	1,155		0	
**1**	Clermont						23	740	-	0	-	26	446		0		26	5,306		0		27	3,069		0	
**5**	Argeles Sur Mer						23	3,080	-	0	-															
**4**	Argeles Sur Mer											25	92		0		24	81		0		21	62		0	
**3**	Toreilles	21	229	-	0	-																				
**Sentinel sites**																										
**France**																										
**6**	Vives						1	7	19/10/2009	0	7															
**10**	Ceret						1	16	19/10/2009	0	16															
**18**	**Reynes**						1	1	19/10/2009	0	1	1	408	11/10/2010	0	408	1	105	18/10/2011	0	105	1	466	**25/09/2012**	**8**	466
**15**	**Ceret**											1	186	11/10/2010	0	186	1	194	18/10/2011	0	194	1	2	**26/09/2012**	**3**	2
**17**	Amélie Les Bains Palalda						1	1	19/10/2009	0	1															
**7**	Taillet						1	1	19/10/2009	0	1															
**21**	**Maureillas** Las Illas											1	0	11/10/2010	0	0	1	109	18/10/2011	0	109	1	10	**25/09/2012**	**11**	10
**20**	Maureillas Las Illas											1	6	11/10/2010	0	6	1	0	18/10/2011	0	0	1	20	25/09/2012	0	20
**14**	**Albere**											1	6	11/10/2010	1	6	1	3	18/10/2011	0	3	1	1	**26/09/2012**	**2**	1

**19**	**Cerbere**						1	0	21/10/2009	0	0	1	5	14/10/2010	0	5	1	3	19/10/2011	0	3	1	9	**24/09/2012**	**3**	9
**16**	Banyuls						1	0	19/10/2009	0	0	1	2	14/10/2010	0	2	1	2	19/10/2011	0	2	1	0	24/09/2012	0	0
**13**	Montesquieu Des Alberes						1	0	20/10/2009	0	0	1	9	13/10/2010	0	9	1	0	18/10/2011	0	0	1	0	26/09/2012	0	0
**11**	Laroque Des Alberes											1	1	13/10/2010	0	1	1	37	17/10/2011	0	37	1	7	24/09/2012	0	7
**12**	**Collioure**											1	1	14/10/2010	0	1	1	14	19/10/2011	0	14	1	103	**25/09/2012**	**1**	103
**8**	Sorede											1	41	13/10/2010	0	41	1	1	19/10/2011	0	1	1	53	24/09/2012	0	53

For *C. imicola* collections, we reported only the data for the maximum abundance collection date. Nb = number. Locations and collection dates in bold are positive sites for *C. imicola* in the Pyrénées-Orientales department.

Supplementary Table 2 | COI and Cytb concatenated genetic diversity and haplotype composition within sample site.

Site	N	H	Hd ± SD	π ± SD	Haplotype composition																														
					H1	H2	H3	H4	H5	H6	H7	H8	H9	H10	H11	H12	H13	H14	H15	H16	H17	H18	H19	H20	H21	H22	H23	H24	H25	H26	H27	H28	H29	H30	H31
Algeria	17	7	0.86 ± 0.05	0.0015 ± 0.0002	5	3	1	3	1	2	2																								
Morocco	14	11	0.95 ± 0.04	0.0020 ± 0.0002		1					3	1	1						2							1	1	1	1	1	1				
Portugal	15	7	0.81 ± 0.09	0.0013 ± 0.0003						2	7		1		2																		1	1	1
Continental Spain	22	11	0.83 ± 0.07	0.0013 ± 0.0002		2					9		1	1	1	1	1	3	1	1	1														
Balearic Islands, Spain	8	3	0.67 ± 0.12	0.0007 ± 0.0001	4						3	1																							
Pyrénées-Orientales, France	17	6	0.75 ± 0.09	0.0010 ± 0.0002	2	3				1	8											2		1											
Var, France	16	3	0.54 ± 0.09	0.0056 ± 0.0001		10				5															1										
Corsica, France	16	6	0.85 ± 0.02	0.0013 ± 0.0001	1	5		3			5											1	1												
Sardinia, Italy	8	3	0.68 ± 0.12	0.0007 ± 0.0002				3		1	4																								

For each site, the number of samples (N), the number of haplotypes (H), haplotype (Hd) and nucleotide (π) diversity and their standard deviations (SD) are indicated.

Supplementary Table 3 | P-values inferred by the Wilcoxon test of bottleneck, F_{IS} values within samples and effective population sizes

Country	Sites	code	IAM	TPM	SMM	F_{IS}
Algeria	Skikda	J	0.0185*	0.5000	0.9355	0.107
	Wilaya de Jijel	I	0.0136*	0.3671	0.8984	0.050
Morocco	Khemisset	H	0.4101	0.9179	0.9951	0.030
	Sidi Yahia El Gharb	G	0.5898	0.9179	0.9970	0.140
Portugal	Castelo Branco	B	0.1250	0.5898	0.9179	0.096
	Beja	A	0.0644	0.5898	0.8984	0.122
Balearic Islands, Spain	Majorca	F	0.1015	0.5898	0.9814	0.097
Continental Spain	Girona	E	0.0644	0.5898	0.1503	0.080
	Toledo	D	0.0097**	0.4101	0.8984	0.010
	Huelva	C	0.0019**	0.0644	0.5449	0.025
Continental France	Pyrénées-Orientales	M	0.0068**	0.1503	0.3671	-0.015
	Roquebrune-sur-Argens	K	0.0019**	0.0097**	0.3261	-0.038
	Bormes-les-Mimosas	L	0.0097**	0.1503	0.4550	0.024
Corsica, France	Figari	N	0.0097**	0.2480	0.8496	0.036
	Pietracorbara	O	0.0019**	0.0644	0.8750	0.112
Sardinia, Italy	San Giovanni Suergiu	P	0.0185*	0.2481	0.7871	0.052

For the bottleneck analysis, p-values correspond to the Wilcoxon unilateral test under each mutation model (Infinite Allele Mutation: IAM, Two-phase model: TPM and Stepwise Mutation Model: SMM). Statistical significance is tested over 10000 replicates (**P < 0.01, *P < 0.05). P-values obtained for the Hardy-Weinberg test were adjusted for multiple comparisons at the nominal level (5%) of 0.00037.

Supplementary Table 4 | Mitochondrial pairwise F_{ST} values between *C. imicola* populations.

	Algeria	Sardinia, Italy	Corsica, France	Pyrénées-Orientales, France	Var, France	Morocco	Portugal	Balearics Island, Spain
Sardinia, Italy	0.03188							
Corsica, France	0.04440	0.00444						
Pyrénées-Orientales, France	0.04421	**0.19543**	0.04354					
Var, France	**0.15972**	**0.34694**	0.10000	0.03702				
Morocco	**0.07275**	**0.11014**	0.04071	0.00905	0.03303			
Portugal	0.06163	**0.07438**	**0.07590**	0.02891	**0.07054**	0.02240		
Balearics Island, Spain	0.02336	**0.28571**	**0.24953**	**0.19914**	**0.47986**	0.12475	**0.19556**	
Continental Spain	**0.12118**	**0.12695**	**0.07619**	**0.05231**	0.05430	0.01229	0.03447	**0.20952**

Significant values are highlighted in bold.

Supplementary Table 5 | Approximate Bayesian computation (ABC) results.

**Description of tested scenarios**	**Posterior probability**	**95% credibility interval**	**Type I error**	**Type II error**
Independent introductions from North Africa into Spain, Sardinia, Corsica and Pyrénées-Orientales	0.07	[0.01-0.13]	0.16	0.06
Introduction from North Africa into Spain, then colonization of Pyrénées-Orientales out from Spain	0.01	[0.00-0.06]	0.18	0.06
Introduction from North Africa into Sardinia, then colonization of Pyrénées-Orientales out from Sardinia	0.29	[0.26-0.32]	0.21	0.09
Introduction from North Africa into Sardinia, then introduction in Corsica out from Sardinia, and then colonization of Pyrénées-Orientales out from Corsica	**0.62**	**[0.60-0.64]**	**0.28**	**0.06**

Type 1 error is the probability of selecting another scenario when the chosen scenario is true. Type 2 error is the mean probability of selecting the chosen scenario when it is false. The selected (most probable) scenario is highlighted in bold (see also Fig. S7).

Supplementary Table 6 | ABC model checking of the most probable scenario (Scenario 4).

summary statistics	observed value	proportion (simulated<observed)	summary statistics	observed value	proportion (simulated<observed)	summary statistics	observed value	proportion (simulated<observed)
NAL_1_1	5.0000	0.6330	V2P_1_1&4	1.3863	0.3315	DAS_1_2&3	0.3945	0.3635
NAL_1_2	4.3333	0.5850	V2P_1_1&5	1.3480	0.3255	DAS_1_2&4	0.3845	0.3985
NAL_1_3	4.6667	0.5440	V2P_1_2&3	1.3258	0.3310	DAS_1_2&5	0.4048	0.3855
NAL_1_4	5.0000	0.5915	V2P_1_2&4	1.3718	0.3355	DAS_1_3&4	0.3984	0.4425
NAL_1_5	4.4444	0.5445	V2P_1_2&5	1.3245	0.3420	DAS_1_3&5	0.4049	0.4220
HET_1_1	0.6101	0.6745	V2P_1_3&4	1.3911	0.3290	DAS_1_4&5	0.3989	0.4565
HET_1_2	0.6113	0.7160	V2P_1_3&5	1.3621	0.3420	DM2_1_1&2	0.0504	0.0805
HET_1_3	0.5909	0.6200	V2P_1_4&5	1.4067	0.3375	DM2_1_1&3	0.0468	0.1135
HET_1_4	0.6074	0.6310	FST_1_1&2	0.0332	0.2965	DM2_1_1&4	0.0354	0.0825
HET_1_5	0.5939	0.6340	FST_1_1&3	0.0352	0.4550	DM2_1_1&5	0.0605	0.1440
VAR_1_1	1.3344	0.3385	FST_1_1&4	0.0261	0.4365	DM2_1_2&3	0.0616	0.2345
VAR_1_2	1.2770	0.3340	FST_1_1&5	0.0320	0.3400	DM2_1_2&4	0.0450	0.0860
VAR_1_3	1.3534	0.3460	FST_1_2&3	0.0146	0.3340	DM2_1_2&5	0.0639	0.3585
VAR_1_4	1.4478	0.3500	FST_1_2&4	0.0159	0.1545	DM2_1_3&4	0.0173	0.0165 (*)
VAR_1_5	1.3576	0.3595	FST_1_2&5	-0.0004	0.2160	DM2_1_3&5	0.0776	0.3590
MGW_1_1	0.9854	0.8330	FST_1_3&4	0.0042	0.0420 (*)	DM2_1_4&5	0.0579	0.1545
MGW_1_2	0.9000	0.6515	FST_1_3&5	0.0119	0.3580			
MGW_1_3	1.0286	0.9160	FST_1_4&5	0.0049	0.0590			
MGW_1_4	0.9712	0.7930	LIK_1_1&2	1.0667	0.5670			
MGW_1_5	0.9677	0.8430	LIK_1_1&3	1.0523	0.6180			
N2P_1_1&2	5.7778	0.6655	LIK_1_1&4	0.9948	0.6055			
N2P_1_1&3	5.7778	0.6080	LIK_1_1&5	1.0554	0.5920			
N2P_1_1&4	5.7778	0.5920	LIK_1_2&1	0.9423	0.4770			
N2P_1_1&5	5.6667	0.6085	LIK_1_2&3	0.8331	0.4720			
N2P_1_2&3	5.0000	0.5110	LIK_1_2&4	0.8503	0.3980			
N2P_1_2&4	5.3333	0.5390	LIK_1_2&5	0.8065	0.5155			
N2P_1_2&5	4.7778	0.5125	LIK_1_3&1	1.0029	0.5390			
N2P_1_3&4	5.5556	0.5470	LIK_1_3&2	0.9266	0.5165			

N2P_1_3&5	5.0000	0.4750	LIK_1_3&4	0.9104	0.4740
N2P_1_4&5	5.4444	0.5520	LIK_1_3&5	0.9307	0.5560
H2P_1_1&2	0.6210	0.6675	LIK_1_4&1	0.9566	0.5355
H2P_1_1&3	0.6120	0.6430	LIK_1_4&2	0.9286	0.4315
H2P_1_1&4	0.6183	0.6590	LIK_1_4&3	0.8742	0.4170
H2P_1_1&5	0.6134	0.6520	LIK_1_4&5	0.9132	0.4460
H2P_1_2&3	0.6038	0.6490	LIK_1_5&1	0.8989	0.4915
H2P_1_2&4	0.6145	0.6485	LIK_1_5&2	0.7852	0.5140
H2P_1_2&5	0.6022	0.6685	LIK_1_5&3	0.8042	0.4940
H2P_1_3&4	0.6010	0.5915	LIK_1_5&4	0.8158	0.4165
H2P_1_3&5	0.5966	0.6160	DAS_1_1&2	0.3730	0.3740
H2P_1_4&5	0.6033	0.5920	DAS_1_1&3	0.3790	0.3770
V2P_1_1&2	1.3115	0.3100	DAS_1_1&4	0.3770	0.3605
V2P_1_1&3	1.3455	0.3230	DAS_1_1&5	0.3826	0.4000

All the summary statistics (used and unused for model selection) were used. For each statistic, the observed value and probability (simulated < observed) are given. For a detail description of the summary statistics, see the DIYABC v.2.0.4 manual. Significant probabilities are represented with an asterisk.

Supplementary Table 7 | Primers characteristics.

Loci	Motif	Forward	Reverse	Allele size range	T (°C)
68	(GT)n	CTTTTCCGTTTCTTTTTATTTCTTT	GTTTCTTTCTGGTCGCGTTGGTTGCTG	99-105	60
12b	(CT)n	TTATGTGTGTATGTTAGCAAGGTCA	GTTTCTTCTTCGGATCAAAGAAATTTTGCC	129-139	50
3b	(AC)n	ATGCGGATGTTTGAAGTG	GTTTCTTTTTTGTGTCTTATTGCCC	154-175	50
31	(CAA)n	TTCTGTTCGGCTGTTGCGTT	GTTTCTTCTTTTTACGTGGTGGTCATTC	152-168	60
41b	(CT)n	GAGGAGGAGGTAGAA	GTTTCTTTCTATTAGTCAATGGTG	155-166	50
35t	(AC)n	TTTGTAAAAGCCAGTTCAACCG	GTTTCTTATCGAACGAAGGAAATAACCAC	171-194	60
88b	(AC)n	TTTGTTCGATTTGTAGTG	GTTTCTTCCTCTCTTTCATTCGC	243-256	50
16	(TG)n	TTGCCTTTGCTTGTTGAGGATG	GTTTCTTTCCTCTTTAAAATCACTGACGTG	292-299	60
88	(CAT)n	GTTGGTGCTTTGTTGTGTTGT	GTTTCTTTTTCTTTTTCTCCTTTTTGTTTCTTTC	344-348	50

Supplementary Table 8 | Prior distributions of demographic, historical and mutation parameters used for ABC inferences.

Parameter description	Parameter	Prior distribution
Stable effective population size	N	Loguniform [1000; 200000]
Time of colonization events in Pyrénées-Orientales	t_p	Uniform [40; 10000]
Time of colonization events in Sardinia, Corsica and Spain	t_s, t_c, t_e	Uniform [140; 10000]
Duration of bottleneck	D_B	Uniform [0; 50]
Founding effective population sizes	N_s	Loguniform [2; 100]
Mean mutation rate	µmicrosatellites	Loguniform [1E-6; 1E-4]
Mean parameter of the geometric distribution of length	P	Uniform [1E-1; 3E-1]
Mean mutation rate for nucleotide instability	SNI	Uniform [1E-8; 1E-4]

Effective population sizes (N) are expressed in number of diploid individuals and times of events (t) in number of generations going back to the past. Conditions among the parameters used during the simulations were ts ≥ tp, te ≥ tp, tc ≥ tp.
